# Effect of Polishing Systems and Accelerated Artificial Aging on the Optical Properties (Color and Translucency) and Surface Roughness of Single-Shade Resin Composites

**DOI:** 10.3390/jfb17080392

**Published:** 2026-08-08

**Authors:** Ayşe İrem Yetiş, Oya Bala

**Affiliations:** Restorative Dentistry Department, Faculty of Dentistry, Gazi University, Ankara 06530, Turkey; oyabala@gazi.edu.tr

**Keywords:** single-shade resin composite, accelerated artificial aging, polishing systems, color change, translucency parameter, surface roughness

## Abstract

This in vitro study aimed to evaluate the effects of different polishing systems and accelerated artificial aging on the optical properties and surface roughness of structurally different single-shade resin composites (SSRCs). Five SSRCs (Omnichroma (OM), Essentia Universal (EU), Admira Fusion X-tra Universal (AFX), Charisma Diamond ONE Shade (CDO), and Zenchroma (ZC)) were evaluated in this study. A total of 250 disc-shaped specimens were randomly assigned to five polishing groups: unpolished control, Sof-lex XT, Clearfil Twist Dia, Enhance-PoGo, and Opti One Step. Measurements were taken after a 24 h incubation in distilled water (37 °C) and repeated after accelerated aging. Changes in color and translucency were calculated using the CIEDE2000 formula. Statistical analyses included the Mann–Whitney U test with Bonferroni correction, the Kruskal–Wallis test, and Spearman’s correlation for interrelationships among ΔE_00_, ΔTP_00_, and ΔSR. CDO and AFX showed lower ΔE_00_ values across all polishing groups (*p* < 0.05). ΔSR differed among resin composites and was generally higher in CDO and AFX (*p* < 0.05). Polishing systems affected both ΔE_00_ and ΔSR within each SSRC group. ΔTP_00_ varied by resin composite, increasing in some groups and decreasing in others. Overall, ΔE_00_, ΔSR, and ΔTP_00_ depended on resin composite type and polishing system. Nano-hybrid resin composites generally demonstrated weaker color changes than microhybrid resin composites, whereas the color stability of the supra-nanofilled composite varied according to the polishing system. Multi-step and diamond-containing polishing systems generally yielded superior optical and surface performance, although their effectiveness depended on resin composite structure. The optical properties of SSRCs, including color, translucency, and surface roughness, were influenced not only by polishing protocols and accelerated artificial aging but also by the structural characteristics of the resin composites. Given the relationships among color, translucency, and surface roughness, careful selection of polishing systems is critical for maintaining the long-term esthetic and functional performance of SSRCs.

## 1. Introduction

Tooth color exhibits a polychromatic nature due to the distinct optical properties of enamel and dentin. Variations in enamel thickness across the cervical, middle, and incisal regions may influence the reflectance of the underlying dentin, thereby contributing to variations in tooth color [1]. Therefore, conventional resin composites developed for restorative applications have been produced in various shades and opacities, including enamel, dentin, opaque, and translucent. To achieve esthetic restorations compatible with natural tooth color, the use of resin composites with different shades and opacities in a layered technique has been recommended [2]. However, this technique is time-consuming and requires a high level of technical sensitivity. In addition, it presents several disadvantages, including interlayer contamination, void formation, and application difficulties, particularly in deep or hard-to-reach cavities [3].

To overcome these limitations, manufacturers have introduced so-called single-shade resin composites (SSRCs). These resin composites are designed to adapt to surrounding tooth structures through the principles of structural color and controlled light scattering, without the use of added pigments. It has been reported that SSRCs can match a wide range of shades in the VITA Classical Shade Guide [4].

The esthetic success of restorative resin composites largely depends on their optical properties, particularly color and translucency. Color is the visual perception resulting from the absorption, reflection, and transmission of light by a resin composite and is commonly quantified using the CIE L*, a*, and b* color coordinates. ΔE_00_ is considered an important parameter indicating the color stability of a resin composite. It is typically evaluated spectrophotometrically by measuring the L*, a*, and b* coordinates and calculating the differences using the CIEDE2000 (ΔE_00_) formula. Owing to its higher correlation with human visual perception, the CIEDE2000 formula has become the preferred method in contemporary studies [5].

Translucency refers to a resin composite’s ability to transmit and scatter light without being fully transparent and plays a critical role in mimicking the optical behavior of natural dental tissues. It is quantitatively assessed using the translucency parameter (TP), which reflects the color difference between a resin composite of a specific thickness and black and white backgrounds and serves as an objective indicator of its light-transmission properties. Over time, changes in this optical characteristic may compromise esthetic integrity. ΔTP_00_ quantitatively reflects changes in light transmission, and evaluating both the current translucency and its temporal changes allows for a more comprehensive assessment of a resin composite’s long-term esthetic performance.

In addition to optical properties, surface characteristics are another critical factor that directly affects the clinical aesthetic performance of resin composite restorations. Surface roughness has been associated with plaque retention, discoloration, gingival inflammation, and secondary caries formation [6]. Therefore, finishing and polishing procedures constitute indispensable steps in restorative applications. Proper finishing and polishing reduce surface roughness, enhance surface gloss and esthetic appearance, and contribute to biological compatibility [7]. Various systems are used to finishing and polishing resin composites, including diamond and carbide burs, aluminum oxide-coated discs, abrasive rubbers, silicon carbide-containing systems, and polishing pastes. Although multi-step aluminum oxide disc systems have been reported to produce smoother surfaces, they are considered relatively time-consuming in the literature [8,9]. Therefore, single- and two-step polishing systems containing micro-sized diamond or silicon carbide particles have been developed to reduce the time required for clinical finishing and polishing procedures [10].

In the oral environment, restorative resin composites undergo aging over time due to exposure to thermal fluctuations, moisture, and light. This process may lead to deterioration in their physical, chemical, and mechanical properties [11]. To simulate these effects in vitro, aging protocols are commonly employed. One study showed that aging resin composites can affect their color, translucency, and surface properties [12]. Accelerated artificial aging is particularly preferred for evaluating optical properties as it enables assessment of long-term changes within a shorter experimental timeframe [13].

Although numerous studies have evaluated the effects of different finishing-polishing systems and aging protocols on the optical properties and surface roughness of conventional resin composites, variations in the properties and application methods of the finishing–polishing systems, as well as differences in aging protocols, continue to create uncertainty regarding the long-term behavior and aesthetic performance of these resin composites. Moreover, limited information is available regarding changes in the color, translucency, and surface roughness of SSRCs, which have gained increasing popularity in recent years, making it difficult to predict the long-term clinical performance of restorations made with these resin composites. Therefore, the aim of this in vitro study was to evaluate the effects of different polishing systems and accelerated artificial aging on the optical properties (color and translucency) and surface roughness of structurally different SSRCs, and to analyze the relationships among these parameters.

The following null hypotheses were tested:

1. The type of single-shade resin composite does not affect the color change (ΔE_00_), translucency change (ΔTP_00_), or surface roughness (ΔSR) after accelerated artificial aging.
2. The polishing system does not affect the color change (ΔE_00_), translucency change (ΔTP_00_), or surface roughness (ΔSR) after accelerated artificial aging.
3. The color change (ΔE_00_), translucency change (ΔTP_00_), and surface roughness (ΔSR) of the tested SSRCs are not correlated.

## 2. Materials and Methods

The SSRCs and polishing systems evaluated in this study, together with their manufacturers and structural characteristics, are presented in Table 1.

### 2.1. Specimen Preparation

A total of 250 disc-shaped specimens (8 mm diameter, 2 mm thickness) were fabricated using plexiglass molds (*n* = 50 per resin composite). The molds were placed on a glass slab covered with a transparent Mylar strip to obtain standardized surfaces. After inserting the SSRCs into the molds, a second Mylar strip and a glass slab were applied to produce smooth, standardized specimen surfaces.

Polymerization of the SSRCs was performed through the glass slab using an LED light-curing unit (D-Light Pro, GC, Tokyo, Japan) for 20 s in accordance with the manufacturer’s instructions. The light tip was positioned perpendicular to the glass surface, in contact with it, and centered over each specimen. The curing unit’s output intensity was 1000 mW/cm^2^ and was verified with a radiometer (Bluephase Meter II, Ivoclar Vivadent, Amherst, NY, USA) before each use.

The specimens prepared for each SSRC were randomly assigned to five groups (*n* = 10 per group). Specimens in Group 1 received no polishing procedure and served as the control group. Specimens in the other groups were polished using Sof-lex XT discs (Group 2), Clearfil Twist Dia (Group 3), Enhance-PoGo (Group 4), or Opti One Step (Group 5).

Polishing procedures were performed by a single operator according to the manufacturer’s instructions, using a low-speed handpiece (10,000 rpm) under dry conditions. Each step was applied for 20 s with light pressure and continuous planar motion. In multi-step systems, specimens were rinsed and air-dried between steps.

After polishing, specimens were stored in distilled water at 37 °C for 24 h. Baseline color and surface roughness measurements were recorded. Subsequently, the specimens were subjected to accelerated artificial aging (Atlas UV 2000, Mount Prospect, IL, USA) for 300 h to simulate approximately one year of clinical service. The aging cycle consisted of 40 min of light exposure at 37 °C, 20 min of light exposure with water spray, 60 min of light exposure only, and 60 min of water spray in darkness.

### 2.2. Color Measurements

Color measurements (L*, a*, and b* values) before and after accelerated artificial aging were performed using a spectrophotometer (VITA Easyshade V, Vita Zahnfabrik, Bad Säckingen, Germany). Measurements were taken under standard D65 illumination conditions, against a white or black-and-white background, with the instrument tip perpendicular to the specimen surface and at the same distance from the specimen each time. All color measurements were performed at the same location and at the same time of day. Measurements were taken from three different points, starting from the center of the specimen surface. Before each measurement, the spectrophotometer was calibrated using the manufacturer’s ceramic calibration block. Measurements were repeated three times for each specimen, and the mean values were recorded.

The following CIEDE2000 color formulas were applied to calculate both the color difference (ΔE_00_) and the translucency parameter (TP_00_). In these formulas, ΔL, ΔC, and ΔH represent the differences in lightness, chroma, and hue between two measurements, or between the specimen over black and white backgrounds in the case of TP_00_. The weighting functions SL, SC, and SH account for the relative contribution of lightness, chroma, and hue to the overall color difference, while the parametric factors KL, KC, and KH serve as correction terms for experimental conditions and were set to 1 in the present study. RT is the rotation function that accounts for interactions between chroma and hue differences in the blue region.

ΔE_00_ = [(ΔL/K_L_·S_L_)^2^ + (ΔC/K_C_·S_C_)^2^ + (ΔH/K_H_·S_H_)^2^ + RT·(ΔC/K_C_·S_C_)·(ΔH/K_H_·S_H_)]½

TP_00_ = [(L′_B_ − L′_W_/K_L_·S_L_)^2^ + (C′_B_ − C′_W_/K_C_·S_C_)^2^ + (H′_B_ − H′_W_/K_H_·S_H_)^2^ + RT·(C′_B_ − C′_W_/K_C_·S_C_)·(H′_B_ − H′_W_/K_H_·S_H_)]½

ΔTP_00_ = TP 2 − TP 1

### 2.3. Surface Roughness Measurements

Surface roughness values were measured before and after accelerated artificial aging using a contact profilometer (Surftest SJ-301, Mitutoyo, Kawasaki, Japan). The device was calibrated before measurements according to the manufacturer’s instructions. Three measurements were taken in the same direction for each specimen, and the mean was recorded as the Ra value. The profilometer parameters were standardized as follows: a cut-off length of 0.8 mm, an evaluation length of 4.0 mm, and a traverse speed of 0.5 mm/s.

For each specimen, ΔSR values were calculated by subtracting the surface roughness values obtained after accelerated artificial aging from those measured before aging.

### 2.4. Scanning Electron Microscopy (SEM) Evaluation

Before and after accelerated artificial aging, one specimen from each group was prepared for SEM analysis. The methodology involved randomly selecting one representative specimen from each experimental group (*n* = 10) for SEM analysis after completing all baseline and post-aging measurements. The specimens were sputter-coated with gold under vacuum for 30 s (LITH-SD800 Sputter Coater, Xiamen Lith Machine Limited, Xiamen, China) and examined using a field emission scanning electron microscope (FE-SEM, Hitachi SU5000, Tokyo, Japan) at ×2000, ×5000, and ×10,000 magnifications. The obtained images were recorded.

### 2.5. Statistical Analysis

Statistical analyses were performed using SPSS version 27.0 (IBM Corp., Armonk, NY, USA). Statistical significance was set at α = 0.05 (95% confidence level). The normality of data distribution was assessed using the Shapiro–Wilk test, which indicated that the data were not normally distributed (*p* < 0.05). Therefore, non-parametric tests were applied.

Intragroup comparisons were performed using the Mann–Whitney U test with Bonferroni correction, while intergroup comparisons were conducted using the Kruskal–Wallis test. Correlations among ΔE_00_, ΔTP_00_, and ΔSR within each group were analyzed using Spearman’s correlation test.

## 3. Results

### 3.1. Color Change (ΔE_00_)

Statistical analyses of ΔE_00_ values obtained for each SSRC and polishing system are presented in Table 2.

Significant differences in ΔE_00_ were observed among SSRCs within each polishing group (*p* < 0.001). CDO consistently exhibited the lowest ΔE_00_ values across all polishing systems. The highest ΔE_00_ values were generally observed for ZC, except with Opti One Step, where OM recorded the highest value. Effect sizes were very large across all polishing groups (η^2^ > 0.80).

Within each SSRC group, ΔE_00_ values were significantly influenced by the polishing system (*p* < 0.001). The highest ΔE_00_ values were obtained with Enhance-Pogo with EU and OM, with the control group with ZC, and with Clearfil Twist Dia with AFX and CDO. The lowest ΔE_00_ values were observed in the control group for EU and CDO, with Sof-lex XT with ZC, Clearfil Twist Dia with OM, and Opti One Step with AFX.

Pairwise comparisons revealed resin composite-dependent differences within the polishing groups. In the control group, ZC and CDO differed significantly from the other composites (*p* < 0.05). All SSRCs differed significantly in the Sof-lex XT group (*p* < 0.05). In the Clearfil Twist Dia group, only EU and AFX did not show a difference (*p* > 0.05), whereas in the Enhance-Pogo group, ZC and OM did not differ significantly (*p* > 0.05). In the Opti One Step group, ZC did not show a significant difference compared with EU and OM (*p* > 0.05). Effect size analysis indicated high η^2^ values for all SSRCs, with the largest effect observed with OM (η^2^ = 0.903) and the smallest with CDO (η^2^ = 0.486).

### 3.2. Change in Translucency Parameters (ΔTP_00_)

The statistical analyses of the TP1, TP2, and ΔTP values obtained are presented in Table 3.

Significant differences in TP1 and TP2 values were observed both among SSRCs and polishing systems (*p* < 0.05). ΔTP_00_ values varied depending on the resin composite and polishing protocol, showing increases in some cases and decreases in others.

In the control group, TP decreased in all SSRCs except ZC, with the greatest decrease observed in AFX. In the Sof-lex XT group, TP increased in EU and CDO, with the greatest increase observed in EU, whereas TP decreased in ZC, OM, and AFX, with the greatest decrease observed in AFX. In the Clearfil Twist Dia group, TP decreased in all SSRCs except OM, with the greatest decrease observed in AFX. In the Enhance-Pogo group, TP generally decreased, with the greatest decrease observed in CDO. In the Opti One Step group, TP increased in ZC and AFX, with the greatest increase observed in ZC, whereas TP decreased in EU, OM, and CDO, with the greatest decrease observed in OM. Regarding the effect size, OM exhibited the largest effect (η^2^ = 0.754), whereas ZC showed the smallest effect (η^2^ = 0.252).

Within polishing systems, the largest ΔTP_00_ effect size was observed in the Enhance-Pogo group (η^2^ = 0.816), whereas the smallest was observed in the Clearfil Twist Dia group (η^2^ = 0.288). When the polishing systems were compared within each SSRC, TP decreased in all polishing groups except Sof-lex XT with EU, with the greatest decrease observed in the Clearfil Twist Dia group. In ZC, TP increased in the control and Opti One Step groups, with the greatest increase observed in the control group, whereas TP decreased in the remaining polishing groups, with the greatest decrease observed in the Enhance-Pogo group. In OM, TP decreased in all polishing groups except Clearfil Twist Dia, with the greatest decrease observed in the control group. In AFX, TP decreased in all polishing groups except Opti One Step, with the greatest decrease observed in the Clearfil Twist Dia group. In CDO, TP decreased in all polishing groups except Sof-lex XT, with the greatest decrease observed in the Enhance-Pogo group. Among the SSRCs, OM exhibited the largest effect size for ΔTP_00_ (η^2^ = 0.754), whereas ZC exhibited the smallest effect size (η^2^ = 0.252).

### 3.3. Surface Roughness Changes (ΔSR)

Statistical analyses of the SR1, SR2, and ΔSR values obtained are presented in Table 4.

When SR1 and SR2 values were analyzed, significant differences were observed both among SSRCs (excluding the control group) and among polishing systems (*p* < 0.05). ΔSR values generally increased significantly after accelerated artificial aging, with the degree depending on the resin composite and polishing protocol.

When the SSRCs were compared within each polishing group, the greatest decrease in surface roughness was observed in EU in the control group, in AFX in the Sof-lex XT and Enhance-Pogo groups, and in ZC in the Opti One Step group. In the Clearfil Twist Dia group, the greatest decrease in surface roughness was observed in AFX, whereas EU showed a different pattern. The smallest decrease in surface roughness was observed in AFX in the control and Opti One Step groups, in ZC in the Sof-lex XT group, and in OM in the Clearfil Twist Dia and Enhance-Pogo groups. Among the polishing systems, the largest effect size for ΔSR was observed in the Sof-lex XT group (η^2^ = 0.457), whereas the smallest effect size was observed in the Opti One Step group (η^2^ = 0.169).

When the polishing systems were compared within each SSRC, the greatest decrease in surface roughness was observed in the control group for EU, the Opti One Step group for ZC, the Sof-lex XT group for OM and AFX, and the Clearfil Twist Dia group for CDO. The smallest decrease in surface roughness was observed in the Sof-lex XT group for EU, in the control and Sof-lex XT groups for ZC, in the Enhance-Pogo group for OM, in the control and Opti One Step groups for AFX, and in the control group for CDO. Among the SSRCs, AFX exhibited the largest effect size for ΔSR (η^2^ = 0.509), whereas CDO exhibited the smallest effect size (η^2^ = 0.028).

### 3.4. Correlation Analysis

**Figure 1:**
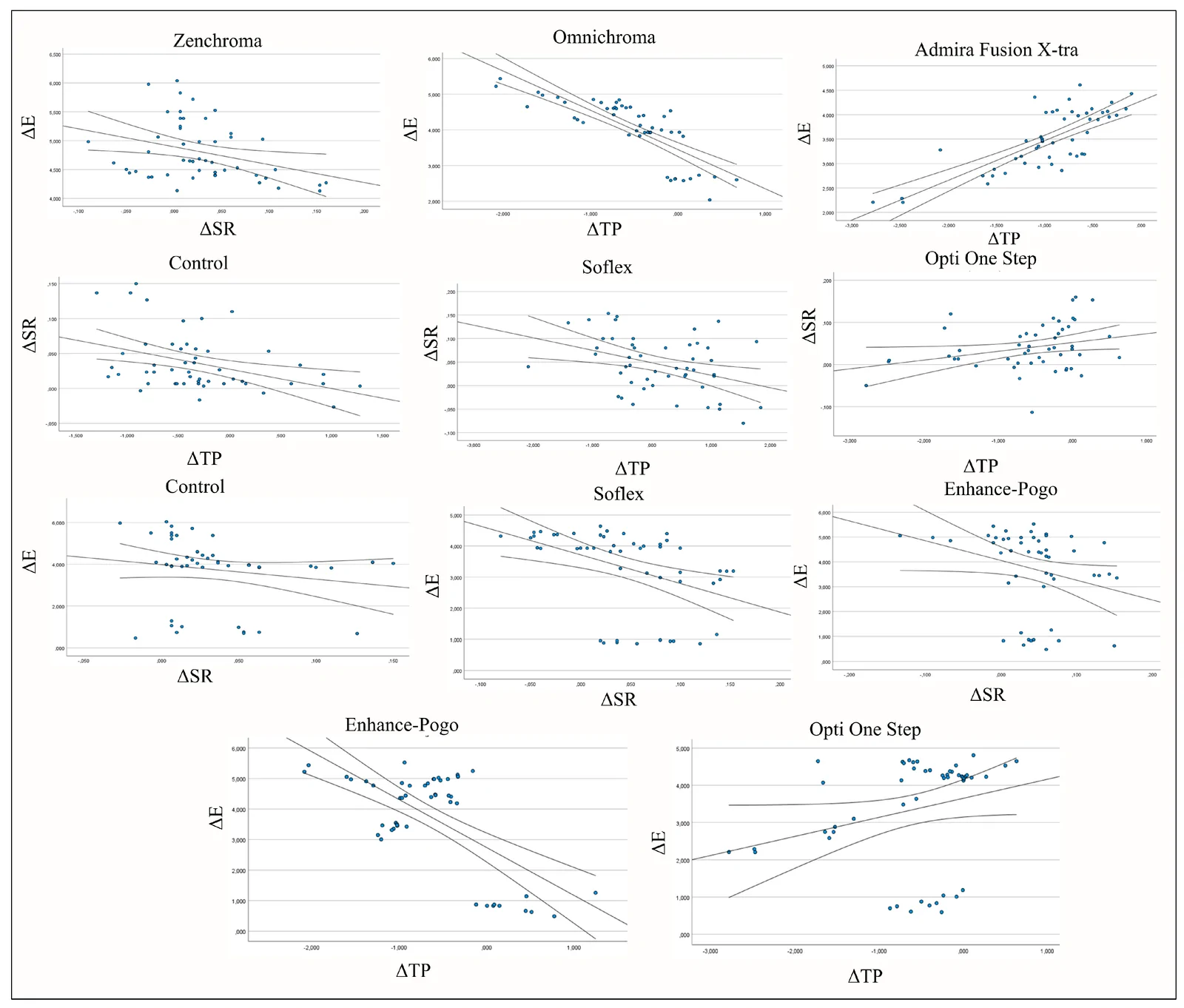
Correlation graphs between the groups tested in the study.

Analysis of correlations among ΔE_00_, ΔTP_00_, and ΔSR revealed material- and polishing-dependent patterns (Figure 1). Among SSRCs, OM showed a strong negative correlation between ΔE_00_ and ΔTP_00_ (rho = −0.852; *p* < 0.001), whereas AFX showed a strong positive correlation (rho = 0.713; *p* < 0.001). In ZC, ΔE_00_ was weakly but significantly negatively correlated with ΔSR (rho = −0.326; *p* = 0.021). No significant correlations were observed in EU or CDO (*p* > 0.05). In polishing-based analyses, significant negative correlations between ΔE_00_ and ΔSR, and between ΔSR and ΔTP_00_, were found in the control and Sof-lex XT groups (*p* < 0.05), whereas ΔSR showed significant negative correlations with both ΔE_00_ and ΔTP_00_ in the Enhance-Pogo group (*p* < 0.05). In the Opti One Step group, significant positive correlations were observed between ΔE_00_ and both ΔSR and ΔTP_00_ (*p* < 0.05). No significant correlations were detected in the Clearfil Twist Dia group.

### 3.5. SEM Findings

**Figure 2:**
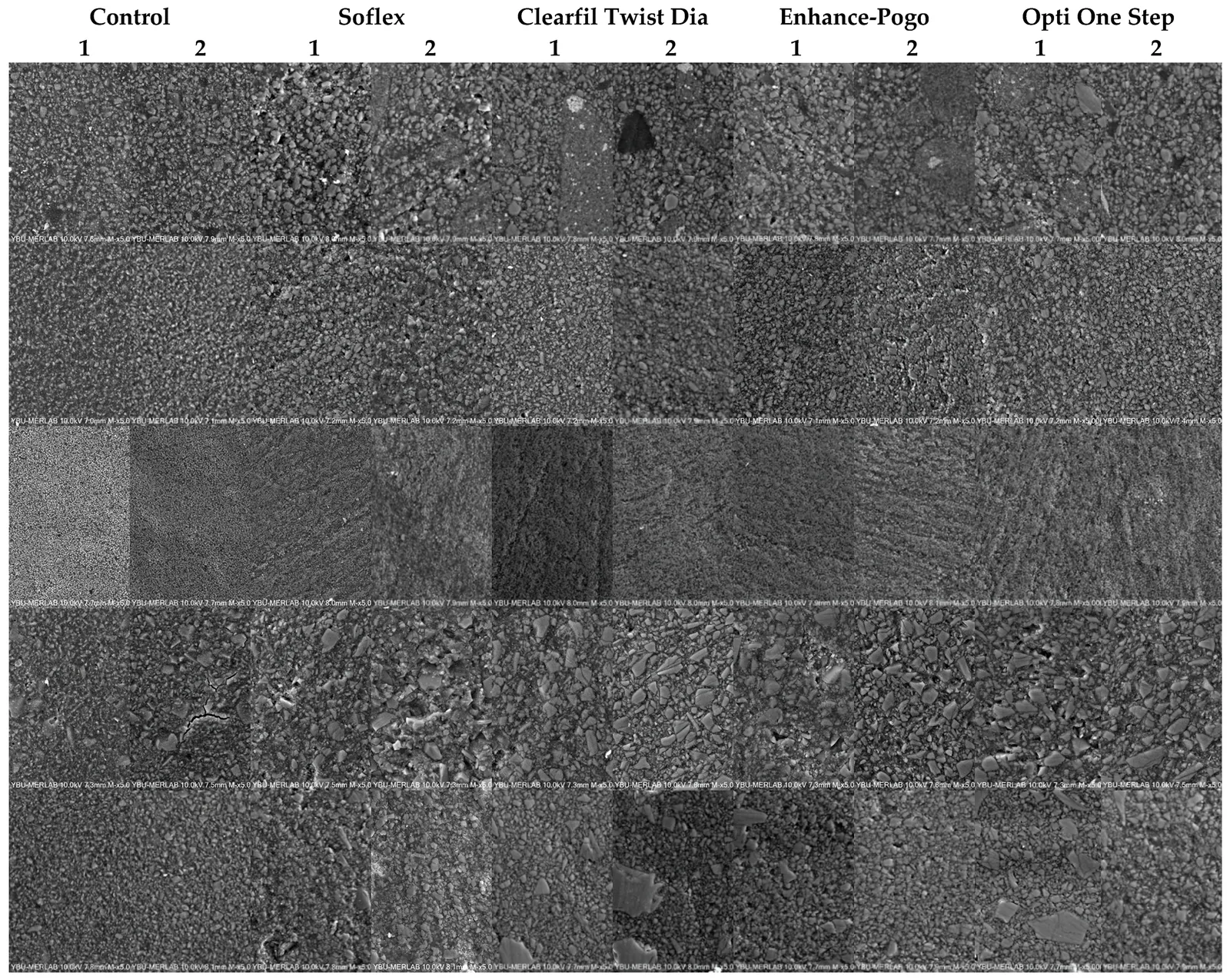
SEM images of the study groups at the baseline (1) and after AAA (2). The rows, from top to bottom, correspond to the EU, ZC, OM, AFX, and CDO resin composites. SEM images were acquired at an accelerating voltage of 10.0 kV using secondary electron (SE) mode at ×5.00k magnification. The scale bar represents 10 μm.

Figure 2 shows the SEM images of the groups tested at baseline and after AAA.

SEM observations before accelerated artificial aging were consistent with the filler types reported by the manufacturers. EU and ZC exhibited microhybrid structures, OM displayed a supra-nanofilled structure, and AFX and CDO demonstrated nano-hybrid characteristics. Following accelerated artificial aging, microhybrid (EU, ZC) and supra-nanofilled (OM) composites showed evidence of filler-matrix degradation and increased surface irregularities. In nano-hybrid resin composites (AFX, CDO), filler-matrix debonding and particle dislodgement were particularly pronounced, indicating structural deterioration.

## 4. Discussion

This in vitro study evaluated the effects of different polishing systems and accelerated artificial aging on the change in the optical properties (color and translucency) and surface roughness of SSRCs.

The findings demonstrated significant differences between the tested SSRCs and polishing systems across all evaluated parameters, and the effect-size analyses indicated that these differences were also clinically relevant. Based on these findings, the study’s first and second null hypotheses were rejected.

Accelerated artificial aging was used in the present study to simulate the long-term degradation of resin composites under standardized laboratory conditions. This protocol allows for the evaluation of optical and surface changes within a relatively short experimental period by exposing resin composites to thermal fluctuations, humidity, and light irradiation that partially mimic intraoral conditions [14]. Although such aging procedures cannot fully reproduce the complex biological, chemical, and mechanical challenges of the oral environment, they are widely accepted as reliable approaches for predicting the long-term performance of restorative resin composites.

According to the CIEDE2000 system, the 50:50 perceptibility threshold is 0.8, whereas the clinical acceptability threshold is 1.8 [15]. In the present study, ΔE_00_ values were mostly below the clinically acceptable limit only in the CDO group. Additionally, the lowest ΔE_00_ values were obtained for CDO across all polishing groups, whereas the highest ΔE_00_ values were generally observed in ZC. These differences may be related not only to the structural characteristics of the tested SSRCs but also to the surface roughness generated after polishing [16]. Increased surface roughness may create retention sites for pigments and chromogenic agents, making resin composites more susceptible to discoloration [17]. Although no definitive clinical threshold for surface roughness (Ra) exists, values above 0.2 µm may increase plaque retention, whereas values exceeding 0.3 µm may become perceptible to patients [18,19]. In the present study, after accelerated artificial aging, Ra values exceeding 0.2 µm were observed only in the AFX group polished with Sof-lex XT and Clearfil Twist Dia, while surface roughness values in all other groups remained within clinically acceptable limits.

Across all polishing groups, relatively lower ΔE_00_ values were generally obtained with CDO, AFX, and OM. However, with respect to ΔSR values, higher roughness changes were particularly observed in CDO and AFX. CDO is a nano-hybrid SSRC containing UDMA, TEGDMA, and TCD-DI-HEA monomers. The rigid cycloaliphatic ring structure of TCD-DI-HEA provides a high cross-link density and low water absorption. These properties may limit hydrolytic degradation, thereby contributing to color stability and helping preserve surface integrity after polishing [20]. In addition, its structure, which contains 5 nm–20 µm barium-aluminum-boron-fluorosilicate glass fillers at 81 wt%, may enhance matrix-filler compatibility, thereby positively influencing both color stability and surface roughness [21]. Accordingly, while low ΔE_00_ values were obtained for CDO, ΔSR values were relatively higher.

AFX, another nano-hybrid resin composite tested in this study, has an ormocer-based matrix and contains silicon dioxide nanoparticles (0.02–0.04 µm) and glass-ceramic fillers at 84 wt%. Despite its high filler content, relatively low ΔE_00_ values were obtained compared with CDO, whereas ΔSR values were generally higher. This finding may be associated with the behavior of the ormocer-based matrix during polishing and aging, which may influence the formation of surface microtopographic irregularities.

In the microhybrid groups EU and ZC, relatively higher ΔE_00_ and ΔSR values were generally observed. The hydrophilic nature of the TEGDMA used in the EU may increase both surface roughness and susceptibility to discoloration [22]. Furthermore, the EU contains pre-polymerized filler particles that are approximately 17 µm in size, which may increase surface irregularities after polishing and, consequently, lead to higher ΔE_00_ and ΔSR values [16,23]. When the microhybrid SSRCs were compared, ZC generally exhibited higher ΔE_00_ and ΔSR values than EU. This difference may be related to the presence of tetramethylene dimethacrylate instead of TEGDMA in ZC as well as its lower filler content (75 wt%) compared with EU.

The ΔE_00_ and ΔSR values obtained for OM varied with the polishing system but were generally high. OM contains uniform, spherical, and homogeneously distributed supra-nano fillers with a particle size of approximately 260 nm. Although this regular filler structure may facilitate smoother polishing, the pigment-free structure may make externally derived colorants more apparent on the surface. This phenomenon may explain the relatively higher color and surface changes observed in OM after accelerated artificial aging [24].

The abrasive type, hardness, and application method of polishing systems may alter the surface microtopography of restorative resin composites, thereby influencing color stability and surface roughness in different ways [25,26]. In this context, Sof-lex XT generally produced lower ΔE_00_ values in all SSRC groups, whereas ΔSR values were higher, particularly in CDO, AFX, and OM. Sof-lex XT is a multi-step polishing system consisting of aluminum oxide-coated discs with different particle sizes (55, 29, 14, and 5 µm) that reduce surface irregularities through a gradual abrasion mechanism [27]. However, in nano-hybrid SSRCs such as CDO and AFX, and supra-nanofilled composites such as OM, the stepwise abrasion mechanism of Sof-lex XT may not always produce the expected smooth surface. While larger aluminum oxide particles remove prominent irregularities, fillers with heterogeneous structures and varying hardness levels may detach from the resin matrix, creating micro-pits [28]. Although finer discs aim to smooth the surface, the effect of this process may remain limited, resulting in increased surface roughness. Interestingly, except for CDO, ΔE_00_ values remained low with Sof-lex XT across all SSRCs, suggesting that the system maintained color stability despite differences in surface roughness.

Clearfil Twist Dia is a two-step polishing system with a spiral design that contains diamond particles of different sizes (medium: 25–35 µm; fine: 4–8 µm). The high hardness of diamond abrasives, combined with the flexibility of the spiral structure, allows for balanced, homogeneous abrasion of both the resin matrix and filler particles during polishing, resulting in a smoother, more uniform surface [29]. In the present study, polishing with Clearfil Twist Dia produced lower ΔE_00_ and ΔSR values, particularly in OM, compared with the other polishing groups, indicating superior performance in terms of both esthetic stability and surface integrity.

Enhance-Pogo is a two-step polishing system containing coarse (40 µm aluminum oxide) and fine (7 µm diamond-polyurethane dimethacrylate) abrasives. This structure may facilitate controlled abrasion of the small, homogeneously distributed fillers in nano-hybrid composites, thereby maintaining relatively low surface roughness and limiting color change [30]. However, color stability varied with the composition of resin composite [31]. While lower ΔE_00_ values were observed in some resin composites, greater color changes were observed in others, particularly in microhybrid and super-nano-filled SSRCs with heterogeneous filler structures. These findings indicate that the influence of Enhance-Pogo on both surface integrity and optical stability is strongly dependent on the characteristics of the resin composite.

Opti One Step is a single-step polishing system containing 0.18 µm diamond particles. The presence of fine and homogeneously distributed abrasives may provide controlled polishing in nano-hybrid composites [32], thereby reducing surface micro-irregularities and contributing to relatively low ΔE_00_ values. However, the single-step design may not be sufficient to completely correct the entire microtopography of nano-hybrid (CDO, AFX) SSRCs. Moreover, the dense distribution of fillers may result in relatively higher ΔSR values after polishing. These findings suggest that although single-step polishing systems may offer advantages in color stability, they may not fully control surface roughness.

Effect size analysis revealed that the highest effect size for ΔE_00_ was observed in the Enhance-Pogo polishing group, whereas the highest effect size for ΔSR was detected in the Sof-lex XT group. Furthermore, the highest effect size for ΔE_00_ among the resin composites was observed in OM, whereas AFX demonstrated the highest effect size for ΔSR. These findings emphasize that both the structural characteristics of the resin composites and the applied polishing systems play a critical role in determining color change and surface roughness.

According to the CIEDE2000 system, the perceptibility threshold for 50:50 TP is 0.62, and the clinical acceptability threshold is 2.62 [33]. In the present study, although the TP values obtained before and after aging for all the tested SSRCs and the polishing groups were above the acceptability limit, the ΔTP_00_ values remained within the threshold limits. The ΔTP_00_ values representing translucency changes after accelerated artificial aging showed increases in some resin composites and decreases in others, depending on the polishing protocol.

Resin composite-based analysis revealed that ΔTP_00_ increased only with Sof-lex XT with EU, while decreases were observed with the other systems, with the most pronounced reduction occurring in the Enhance-Pogo group. In ZC, increases were detected with Clearfil Twist Dia and Enhance-Pogo, with the highest increase observed in the control group and the greatest decrease in the Enhance-Pogo group. In OM, ΔTP_00_ increased only in the Clearfil Twist Dia group, whereas decreases were observed with the other systems. In AFX, ΔTP_00_ decreased across all polishing groups, with the greatest reduction in the Sof-lex XT group. In CDO, ΔTP_00_ increased with Sof-lex XT and Enhance-Pogo, whereas the greatest decrease was observed with Opti One Step.

The observed variations in ΔTP_00_ values indicate that translucency is strongly influenced by resin composite composition, monomer structure, filler type and size, and the refractive-index compatibility between the resin matrix and filler particles [34]. For instance, the higher translucency of Bis-GMA compared with UDMA and TEGDMA, increased light scattering caused by larger filler particles, and the presence of radiopaque agents and pigments may explain these differences [34,35]. In addition, abrasive characteristics, particle size, and the application method of the polishing system may influence ΔTP_00_, leading to increased translucency in some resin composites and decreased translucency in others. These changes may directly affect the esthetic appearance of restorations: excessive translucency may increase the visibility of underlying structures and result in color mismatch, whereas increased opacity may limit light transmission and reduce the natural appearance of the tooth [36].

When the relationships among ΔE_00_, ΔSR, and ΔTP_00_ were evaluated, resin-composite-specific responses were observed in each SSRC group. In EU and CDO, no significant relationships were detected among these parameters, suggesting that color change, surface roughness, and translucency behaved independently. In ZC, a significant negative correlation was observed only between ΔE_00_ and ΔSR, suggesting that surface roughness may have a limited influence on color change. In OM, a strong negative correlation was observed between ΔE_00_ and ΔTP_00_, indicating that increased color change was associated with reduced translucency. In contrast, AFX demonstrated a positive, highly significant relationship between ΔE_00_ and ΔTP_00_, suggesting that translucency increased with increasing color change.

When the relationships among ΔE_00_, ΔSR, and ΔTP_00_ were analyzed by the polishing system, distinct patterns emerged. In the control and Sof-lex XT groups, significant negative correlations were observed between ΔE_00_ and ΔSR as well as between ΔSR and ΔTP_00_, suggesting that increased surface roughness may limit both color change and translucency. Similarly, negative relationships between ΔSR and both ΔE_00_ and ΔTP_00_ were observed in the Enhance-Pogo group, indicating that surface microtopography may play an important role in both color stability and light transmission. In contrast, in the Opti One Step group, significant positive correlations were observed among ΔE_00_, ΔSR, and ΔTP_00_, indicating that increases in color change were accompanied by increases in both surface roughness and translucency. Based on these findings, the third null hypothesis was partially rejected.

Despite the comprehensive evaluation of color stability, translucency, and surface roughness, several limitations of the present study should be considered. First, the in vitro design cannot fully replicate the complex conditions of the oral environment, where factors such as salivary enzymes, dietary pigments, biofilm accumulation, and mechanical wear may further influence the optical and surface properties of resin composites. In addition, only a limited number of SSRCs and polishing systems were evaluated. Therefore, the results should be interpreted within the limitations of the experimental conditions.

Overall, the present findings indicate that both the structural properties of resin composites, including monomer composition, filler type, and particle size, and the applied polishing systems play a decisive role in determining color stability, translucency, and surface roughness after aging. Nano-hybrid (CDO, AFX) and supra-nanofilled (OM) SSRCs generally demonstrated weaker color change due to their more homogeneous filler structures. In contrast, variations in surface roughness were largely influenced by the abrasive properties and application protocols of the polishing systems. From a clinical perspective, these findings suggest that the long-term esthetic performance of SSRC restorations depends not only on the structural properties of the resin composite but also on the polishing protocol used after finishing. Therefore, selecting polishing systems compatible with the composite’s structural characteristics may help maintain color stability, preserve translucency, and minimize surface roughness over time, ultimately improving the long-term esthetic and functional success of resin composite restorations.

## 5. Conclusions

Within the limitations of this in vitro study, the following conclusions can be drawn:

1. Both the structural characteristics of the tested single-shade resin composites (SSRCs) and the polishing systems affected color stability, translucency, and surface roughness after accelerated artificial aging.
2. The effect of polishing systems on optical and surface properties was dependent on the structural characteristics of the SSRCs.
3. Nano-hybrid resin composites (CDO and AFX) generally demonstrated weaker color changes than the microhybrid resin composites (ZC and EU), whereas the color stability of the supra-nanofilled resin composite (OM) was more dependent on the polishing system.
4. Although translucency generally decreased in all tested SSRCs, the greatest influence of resin composite type on translucency change was observed with the two-step Enhance-PoGo polishing system, while the highest translucency change was recorded for the supra-nanofilled resin composite (OM).
5. Regarding surface roughness, the influence of SSRC type was most pronounced with the multi-step Sof-Lex polishing system, and the greatest increase in surface roughness was also observed for the supra-nanofilled resin composite (OM).

## Figures and Tables

**Table 1:** The SSRCs and polishing systems.

Resin Composite	Type	Composition	Filler Content (% *w*/*v*)	Lot No.
**Essentia Universal (EU)** **(GC, Tokyo, Japan)**	Micro-hybrid	**Resin matrix:** Bis-GMA, UDMA, TEGDMA, Bis-MEPP, Bis-EMA **Filler content:** Pre-polymerized fillers (17 μm), strontium glass (400 nm), lanthanide fluoride (100 nm), silica (16 nm), silicon glass (850 nm)	81/65	2405131
**Zenchroma (ZC)** **(President, München, Germany)**	Micro-hybrid	**Resin matrix:** Bis-GMA, UDMA, tetrametilen dimetakrilat **Filler content:** Glass powder, SiO_2_	75/53	2024002112
**Omnichroma (OM)** **(Tokuyama, Tokyo, Japan)**	Supra nano-filled	**Resin matrix:** UDMA, TEGDMA **Filler content:** Spherical SiO_2,_- ZrO_2_ filler (260 nm)	79/68	156E84
**Admira Fusion X-tra (AFX)** **(VOCO, Cuxhaven, Germany)**	Nano-hybrid	**Resin matrix:** Ormocer, Bis-GMA, UDMA, TEGDMA, HEMA **Filler content:** Silicon dioxide nanoparticles (0.02–0.04 μm), glass-ceramic filler	84/69	2501098
**Charisma Diamond One (CDO)** **(Kulzer, Wehrheim, Germany)**	Nano-hybrid	**Resin matrix:** UDMA, TEGDMA, TCD-matrix **Filler content:** Silica, barium, aluminum borofluorosilicate glass (5 nm–20 μm), titanium dioxide	81/64	N010209
**Polishing System**	**Composition**	**Type**	**Application Step**	**Lot No.**
**Sof-Lex XT 2382** **(Solventum, Eagan, MN, USA)**	Aluminum oxide Coarse (55 µm) Medium (29 µm) Fine (14 µm) Super fine (5 µm)	Disc	Four step	10579343
**Clearfil Twist Dia** **(Kuraray, Tokyo, Japan)**	Diamond particles Medium (25–35 µm) Fine (4–8 µm)	Spiral	Two step	504658
**Enhance-Pogo** **(Dentsply, Charlotte, NC, USA)**	Enhance (Aluminum oxide -40 µm) PoGo (Diamond, polyurethane dimethacrylate −7 µm)	Disc	Two step	00144292
**Opti One Step** **(Kerr, Brea, CA, USA)**	Diamond and silicon carbide particles, silicon dioxide particles (0.18 μm)	Disc	One step	7844470

**Table 2:** Statistical analysis of color changes (ΔE_00_) obtained from the groups used in the study.

Polishing Groups	Color Change (ΔE_00_)										* *p*–Values/η^2^
	SSRCs										
	EU		ZC		OM		AFX		CDO		
	Median	Min–Max	Median	Min–Max	Median	Min–Max	Median	Min–Max	Median	Min–Max	
**Control**	3.90 ^abABC^	3.82–4.10	5.50 ^acdeABCD^	5.21–6.04	4.24 ^cfABC^	3.85–4.60	4.01 ^dgABC^	3.90–4.43	0.74 ^befgA^	0.46–1.28	<0.001 */0.83
**Sof–Lex XT**	4.12 ^abcdD^	3.91–4.32	4.40 ^aefgAE^	4.35–4.64	3.93 ^behkDEF^	3.82–4.05	3.14 ^cfhlAD^	2.80–3.27	0.93 ^dgklB^	0.86–1.15	<0.001 */0.92
**Clearfil Twist Dia**	4.27 ^abcAE^	3.99–4.34	4.55 ^adefBF^	4.35–4.68	2.62 ^bdghADGH^	2.04–2.73	4.09 ^egkDEF^	3.98–4.61	1.75 ^cfhkABCD^	1.43–2.35	<0.001 */0.89
**Enhance–Pogo**	4.40 ^abcdBDE^	4.18–4.47	5.04 ^aefCEFG^	4.94–5.52	4.88 ^bghBEGK^	4.76–5.43	3.43 ^cegkBE^	3.00–3.54	0.83 ^dfhkC^	0.48–1.25	<0.001 */0.92
**Opti One Step**	4.22 ^abcC^	4.07–4.36	4.27 ^deDG^	4.13–4.80	4.60 ^afgCFHK^	4.37–4.67	2.74 ^bdfhCF^	2.20–3.63	0.80 ^ceghD^	0.59–1.18	<0.001 */0.84
** *p*–values/η^2^	<0.001 **/0.54		<0.001 **/0.78		<0.001 **/0.90		<0.001 **/0.79		<0.001 **/0.48		

* The same lowercase letters in the same row within each polishing group indicate a statistically significant difference in color change among SSRCs (*p* < 0.05). ** The same uppercase letters in the same column within each SSRC indicate a statistically significant difference in color change among polishing groups (*p* < 0.05). η^2^—represents the effect size for the Kruskal–Wallis test.

**Table 3:** Statistical analysis of changes in the translucency parameters (TP1, TP2, and ΔTP_00_) of the groups included in the study.

Polishing Groups	TP	TP Change					
		SSRCS					
		EU	ZC	OM	AFX	CDO	* *p*–Values/η^2^
		Median (Min–Max)	Median (Min–Max)	Median (Min–Max)	Median (Min–Max)	Median (Min–Max)	
**Control**	**TP1**	7.84 ^abcdABC^ (7.08–7.97)	7.26 ^aefAB^ (6.78–7.77)	9.24 ^beghAB^ (7.98–10.28)	9.25 ^cgkA^ (9.13–9.34)	6.37 ^dfhkABCD^ (3.9–7.41)	<0.001 */0.93
	**TP2**	7.28 ^abcdABC^ (6.02–7.60)	8.18 ^aefg^ (7.96–8.61)	9.09 ^behkAB^ (8.73–9.81)	8.60 ^cfhlABC^ (8.34–8.95)	6.19 ^dgklA^ 2.91–7.44	<0.001 */0.95
	**ΔTP**	−0.43 (−1.29)–(−0.02)	0.92 (0.23–1.83)	−0.26 (−0.89–1.07)	−0.58 (−0.98)–(−0.34)	−0.15 (−1.66–0.09)	<0.001 */0.49
**Sof–Lex**	**TP1**	9.80 ^abcdDEF^ (9.02–10.53)	10.07 ^aefgCD^ (9.11–10.37)	11.06 ^behkCD^ (10.68–11.23)	11.00 ^cfhl^ (10.91–11.06)	10.27 ^dgklAE^ (9.78–11.47)	<0.001 */0.90
	**TP2**	10.26 ^abcADEF^ (10.09–10.54)	9.87 ^adefA^ (9.57–11.32)	10.74 ^bdghACDE^ (10.14–11.93)	10.54 ^egkAD^ (10.08–10.75)	10.44 ^cfhkB^ (9.19–10.73)	<0.001 */0.90
	**ΔTP**	0.64 (−0.30–1.27)	−0.23 (−0.56–1.15)	−0.25 (−1.07–1.00)	−0.48 (−0.93)–(−0.16)	0.02 (−0.73–0.63)	<0.001 */0.71
**Clearfil Twist Dia**	**TP1**	12.86 ^abADG^ (12.67–13.24)	12.91 ^acdeACE^ (12.75–13.04)	13.44 ^bcfgACEF^ (13.20–13.55)	13.21 ^dfh^ (13.05–13.45)	12.78 ^eghBFG^ (11.58–12.96)	<0.001 */0.87
	**TP2**	12.13 ^abcBDGH^ (11.59–12.56)	12.60 ^adefA^ (12.46–12.95)	13.54 ^bdghBEFG^ (13.28–14.09)	11.96 ^cegkEF^ (11.02–12.47)	12.25 ^fhkABCD^ (9.85–12.63)	<0.001 */0.87
	**ΔTP**	−0.73 (−1.18)–(−0.33)	−0.30 (−0.43–0.05)	0.10 (−0.12–0.67)	−1.33 (−2.08)–(−0.67)	−0.57 (−1.72–0.08)	0.006 */0.28
**Enhance–Pogo**	**TP1**	9.238 ^abcdBEH^ (9.025–9.439)	8.77 ^aefgBDF^ (8.68–10.46)	8.92 ^behkBDEG^ (8.71–9.64)	8.86 ^cfhlA^ (8.79–8.93)	8.90 ^dgklCFH^ (8.86–10.02)	<0.001 */0.94
	**TP2**	8.775 ^abcdCEGK^ (8.52–9.09)	8.04 ^aef^ (7.856–8.41)	8.27 ^beghDF^ (7.99–8.97)	7.78 ^cfgkBDE^ (7.58–7.91)	7.36 ^dhkC^ (6.85–8.32)	<0.001 */0.89
	**ΔTP**	−0.37 (−0.87)–(−0.09)	−0.87 (−2.08)–(−0.58)	−0.68 (−1.10)–(−0.15)	−1.05 (−1.24)–(−0.91)	−1.56 (−2.78)–(−0.55)	<0.001 */0.81
**Opti One Step**	**TP1**	11.52 ^abcdCFGH^ (11.23–12.08)	10.54 ^aefgEF^ (9.95–10.71)	9.95 ^behkFG^ (9.65–11.35)	10.48 ^cfhl^ (10.27–11.84)	11.90 ^dgklDEGH^ (11.69–12.05)	<0.001 */0.93
	**TP2**	11.26 ^abcdFHK^ (10.64–11.75)	11.20 ^aefg^ (11.07–12.20)	9.58 ^behkEG^ (8.50–11.08)	10.70 ^cfhlCF^ (10.31–12.29)	11.54 ^dgklD^ (11.08–11.92)	<0.001 */0.89
	**ΔTP**	−0.25 (−1.04–0.38)	0.70 (0.41–1.76)	−0.85 (−1.28–1.24)	0.29 (−0.12–1.24)	−0.35 (−0.87–0.005)	<0.001 */0.55
** *p*–values for TP1/η^2^		<0.001 */0.84	<0.001 */0.65	<0.001 */0.72	0.006 */0.23	<0.001 */0.74	
** *p*–values for TP2/η^2^		<0.001 */0.89	0.001 */0.31	<0.001 */0.72	<0.001 */0.73	<0.001 */0.46	
** *p*–values for ΔTP/η^2^		<0.001 */0.54	0.004 */0.25	<0.001 */0.75	<0.001 */0.52	<0.001 */0.46	

* The same lowercase letters in the same row within each polishing group indicate a statistically significant difference in TP1, TP2, or ΔTP among SSRCs (*p* < 0.05). ** The same uppercase letters in the same column within each SSRC indicate a statistically significant difference in TP1, TP2, or ΔTP among polishing groups (*p* < 0.05). η^2^—represents the effect size for the Kruskal–Wallis test.

**Table 4:** Statistical analysis of changes in the surface roughness (SR1, SR2, and ΔSR) of the groups included in the study.

Polishing Groups	SR	Surface Roughness Change					
		SSRCS					
		EU	ZC	OM	AFX	CDO	* *p*–Values/η^2^
		Median (Min–Max)	Median (Min–Max)	Median (Min–Max)	Median (Min–Max)	Median (Min–Max)	
**Control**	**SR1**	0.02 ^ABCD^ (0.01–0.04)	0.01 ^ABCD^ (0.01–0.04)	0.01 ^ABCD^ (0.01–0.03)	0.02 ^ABCD^ (0.01–0.02)	0.02 ^ABC^ (0.01–0.04)	0.107/0.152
	**SR2**	0.13 ^ab^ (0.03–0.17)	0.02 ^ac^ (0.01–0.05)	0.05 ^c^ (0.04–0.07)	0.02 ^b^ (0.01–0.07)	0.04 (0.02–0.14)	<0.001 */0.474
	**Δ** **SR**	0.09 (0.01–0.15)	0.007 (−0.02–0.03)	0.02 (0.01–0.05)	0.008 (−0.003–0.05)	0.03 (−0.01–0.12)	<0.001 */0.447
**Sof–Lex**	**SR1**	0.12 ^A^ (0.04–0.24)	0.12 ^aAE^ (0.07–0.23)	0.06 ^abA^ (0.04–0.13)	0.13 ^bcAE^ (0.07–0.17)	0.09 ^cADE^ (0.07–0.12)	<0.001 */0.360
	**SR2**	0.15 ^a^ (0.04–0.19)	0.13 ^b^ (0.08–0.23)	0.11 ^cd^ (0.06–0.15)	0.25 ^abce^ (0.14–0.31)	0.17 ^de^ (0.11–0.21)	<0.001 */0.436
	**Δ** **SR**	0.01 (−0.08–0.08)	0.007 (−0.04–0.08)	0.03 (−0.04–0.10)	0.11 (0.04–0.15)	0.06 (0.02–0.13)	<0.001 */0.457
**Clearfil Twist Dia**	**SR1**	0.10 ^aB^ (0.04–0.15)	0.09 ^bBF^ (0.05–0.15)	0.10 ^B^ (0.04–0.18)	0.10 ^cB^ (0.03–0.17)	0.04 ^abcD^ (0.01–0.06)	<0.001 */0.359
	**SR2**	0.11 ^a^ (0.06–0.14)	0.13 (0.08–0.19)	0.10 ^bc^ (0.05–0.17)	0.22 ^abd^ (0.07–0.28)	0.09 ^cd^ (0.06–0.17)	0.004 */0.311
	**Δ** **SR**	−0.005 (−0.06–0.09)	0.03 (−0.06–0.10)	0.007 (−0.03–0.03)	0.09 (0.007–0.22)	0.07 (0.01–0.12)	<0.001 */0.424
**Enhance–Pogo**	**SR1**	0.11 ^aC^ (0.05–0.14)	0.06 ^C^ (0.05–0.15)	0.10 ^C^ (0.03–0.19)	0.08 ^CE^ (0.04–0.11)	0.05 ^aBE^ (0.03–0.09)	0.033 */0.214
	**SR2**	0.15 (0.10–0.20)	0.10 (0.06–0.15)	0.10 (0.04–0.17)	0.15 (0.07–0.23)	0.10 (0.09–0.19)	0.007 */0.288
	**Δ** **SR**	0.05 (0.00–0.09)	0.03 (−0.09–0.09)	0.00 (−0.13–0.13)	0.06 (0.01–0.15)	0.04 (0.003–0.15)	0.016 */0.251
**Opti One Step**	**SR1**	0.08 ^aD^ (0.06–0.13)	0.05 ^abDEF^ (0.02–0.09)	0.05 ^D^ (0.02–0.18)	0.12 ^D^ (0.04–0.15)	0.06 ^blC^ (0.02–0.13)	0.002 */0.358
	**SR2**	0.14 ^a^ (0.08–0.17)	0.14 (0.04–0.18)	0.06 ^ab^ (0.05–0.13)	0.13 ^b^ (0.08–0.16)	0.11 (0.08–0.20)	0.010 */0.270
	**Δ** **SR**	0.04 (−0.01–0.10)	0.07 (−0.02–0.16)	0.02 (−0.11–0.08)	0.008 (−0.05–0.12)	0.05 (−0.01–0.11)	0.082/0.169
** *p*–values for SR1/η^2^		<0.001 */0.47	<0.001 */0.67	<0.001 */0.51	<0.001 */0.59	<0.001 */0.54	
** *p*–values for SR2/η^2^		0.061 */0.11	<0.001 */0.44	0.001 */0.34	<0.001 */0.66	<0.001 */0.44	
** *p*–values for ΔSR/η^2^		0.007 */0.22	0.039 */0.13	0.09/0.08	<0.001 */0.50	0.260/0.02	

* The same lowercase letters in the same row within each polishing group indicate a statistically significant difference in SR1, SR2_,_ or ΔSR among SSRCs (*p* < 0.05). ** The same uppercase letters in the same column within each SSRC indicate a statistically significant difference in SR1, SR2,, or ΔSR among polishing groups (*p* < 0.05). η^2^—represents the effect size for the Kruskal–Wallis test.

## Data Availability

The original contributions presented in the study are included in the article, further inquiries can be directed to the corresponding author.
